# A network psychometric validation of the Children Oral Health-Related Quality of Life (COHQoL) questionnaire among Aboriginal and/or Torres Strait Islander children

**DOI:** 10.1371/journal.pone.0273373

**Published:** 2022-08-18

**Authors:** Pedro Henrique Ribeiro Santiago, Marko Milosevic, Xiangqun Ju, Wendy Cheung, Dandara Haag, Lisa Jamieson

**Affiliations:** 1 Australian Research Centre for Population Oral Health (ARCPOH), Adelaide Dental School, The University of Adelaide, Adelaide, Australia; 2 School of Public Health, The University of Adelaide, Adelaide, Australia; Macquarie University, AUSTRALIA

## Abstract

In Australia, research evidence has shown that Aboriginal and/or Torres Strait Islander children experience a higher burden of oral health diseases compared to other non-Indigenous children. The impact of oral health diseases on children’s functional and psychosocial outcomes led to the development of several instruments to evaluate child oral health-related quality of life (COHQoL), such as the Parental-Caregiver Perception Questionnaire (P-CPQ) and the Family Impact Scale (FIS). However, the psychometric properties of these instruments have been evaluated only in Western cultures and have not been investigated for Aboriginal children in Australia. The current study aimed to examine the psychometric properties of the short-forms P-CPQ and FIS for Aboriginal and/or Torres Strait Islander children aged 2–3 years. Data were collected from the South Australian Aboriginal Birth Cohort (SAABC), including 270 Aboriginal children aged 2–3 years. Network psychometric models were used to investigate dimensionality, item redundancy, structural consistency and item stability, model fit, internal consistency reliability and criterion validity. We propose an instrument named Aboriginal Children’s Oral Health-Related Quality of Life Questionnaire (A-COHQoL). Our findings indicated that, after the exclusion of four problematic items, the A-COHQoL showed a three-dimensional structure (“Parent/Family Activities”, “COHQoL” and “Family Conflict”) with good model fit and reliability. The A-COHQoL is a psychometrically robust and sensitive instrument that is readily available for Aboriginal and/or Torres Strait Islander children aged 2–3 years in Australia and can be adapted in the future for Indigenous child groups in other countries.

## Introduction

Oral health is an integral part of general health. Oral diseases are an important public health problem due to their prevalence, expense associated with treatment and impact on individuals and societies. Oral diseases emerge early in life, with untreated dental caries in deciduous teeth being the most prevalent chronic condition among children (10th most prevalent condition overall), affecting 531 million children worldwide [1]. Findings from the Australian Burden of Disease Study 2015 reported that, among all children aged 5–14 years, dental caries was the third leading cause of total disease burden, after asthma and mental health disorders [2].

The distribution of oral diseases is characterized by pervasive inequalities primarily underpinned by the social determinants of health, such as social class and racial background. The Australian 2012–14 National Child Oral Health Study (NCOHS) revealed that Indigenous (those identifying as Aboriginal and/or Torres Strait Islander) children in Australia aged 5 to 10 years had, on average, almost three times the mean number of decayed, missing and filled tooth surfaces (dmfs) than non-Indigenous children (3.4 *vs* 1.2) [3]. The prevalence of untreated dental caries in the primary dentition was 70% higher among Indigenous children relative to non-Indigenous children (40% *vs* 26%), suggesting that Indigenous Australian children not only experience greater levels of disease but are also less likely to access dental treatment.

Empirical research has provided compelling evidence of the impacts of oral conditions on the daily life of children and their families. It is now recognized that the impacts associated with poor oral in childhood are extensive, affecting children and their families, with Indigenous children carrying a larger share of the disease burden and related impacts. This means that Indigenous children are more likely to be exposed to the consequences of poor oral health, which may involve toothache, difficulties concentrating, school absenteeism, poor academic performance, increased likelihood of general anaesthesia and dissatisfaction with dental appearance [4–8].

Increasing interest in the functional and psychosocial impacts related to oral diseases among children has led to the development of specific instruments to assess child oral health-related quality of life (COHQoL). These assessments are powerful research tools and may work as a guide for clinical practice and policymaking. From a research point of view, they allow for a more nuanced understanding of the oral health-related impacts on individuals’ well-being [9]. Furthermore, COHQoL findings can shed light on the pathways through which oral health may affect individuals’ well-being, contributing to better targeting of specific intervention strategies [10]. Assessment of COHQoL may also be useful for surveillance and evaluation of healthcare interventions, supporting the development of evidence-based public health strategies. When employed in clinical settings, these measurements may work as a useful communication tool for identifying and prioritizing patient problems and preferences.

Despite the disproportionate oral disease burden experienced by Indigenous Australian children, to the best of our knowledge, no studies assessing the psychometric properties of COHQoL instruments have been carried out in this population. Most COHQoL instruments have been evaluated in Western settings in children from the general population. This gap means we know little about the complex interplay of symptoms and functional impacts related to oral diseases that may affect the quality of life of Indigenous children. Considering the disproportionate rates of dental disease and stressful life events experienced by Indigenous Australians and their children, it is crucial to develop or adapt instruments that can measure the quality of life related to oral health and are culturally valid for this group. Without this knowledge, it becomes difficult for policymakers to develop and implement interventions to improve outcomes for Indigenous children who experience dental disease.

The Parental-Caregiver Perceptions Questionnaire (CPQ), an instrument originally developed to designed to measure parental/caregiver perceptions of the oral health-related quality of life of children [11], and the Family Impact Scale (FIS), an instrument developed to assess the effect of oral conditions on family functioning [12], have been developed in Canada and cross-culturally validated in multiple countries, including Brazil [13], France [14], China [15], Peru [16], the UK [17] and USA [18]. Thomson and colleagues developed the short form P-CPQ and FIS and found that these measures had adequate reliability and validity, and acceptable responsiveness in New Zealand’s context [19]. The advantage of short measures over the full versions is that they induce a lower response burden on respondents, particularly when applied as part of an extensive questionnaire assessing multiple aspects of oral health and well-being. This is important considering the key role of COHQoL assessments in surveillance systems. The present study aimed to evaluate the psychometric properties of the caregiver-informant short-form P-CPQ and FIS versions for Indigenous Australian children aged 2 to 3 years in South Australia.

## Methods

### Design and setting

Data were collected from the South Australian Aboriginal Birth Cohort (SAABC), a prospective longitudinal birth cohort study which initiated as a 2-arm parallel, outcome assessor-blinded, randomised controlled trial that aimed to assess if an intervention involving dental care to mothers during pregnancy, application of fluoride varnish to the teeth of children, anticipatory guidance and motivational interviewing reduced prevalence of dental disease among Indigenous children in South Australia. The intervention took place during pregnancy and when children were aged 6, 12 and 18 months for the intervention group, and when children were aged 24 months, 30 months and 36 months for the delayed intervention group [20].

Eligibility involved being pregnant with an Aboriginal and/or Torres Strait Islander child between February 2010 and May 2011 and residing in South Australia. Recruitment was through the antenatal clinics of South Australian Aboriginal Community Controlled Health Organisations and hospitals. The SAABC received ethical approval from the University of Adelaide Human Research Ethics Committee, the Aboriginal Health Council of South Australia, the Government of South Australia and the Human Research Ethics Committees of three participating South Australian hospitals [11]. All procedures performed in the SAABC studies were in accordance with the ethical standards of the institutional and/or national research committee and with the 1964 Helsinki declaration and its later amendments or comparable ethical standards. All participants provided written informed consent. For purposes of this study, data from the 2-year follow-up was used [20]. The SAABC 2-year follow-up occurred between January 2013 and November 2014 and the data was analysed retrospectively. The SAABC is an ongoing birth cohort study and, in addition to the 2-, 3-, 5- and 7-year-old follow-ups, the families and children are currently participating in the 9-year-old follow-up. For more information on the SAABC, please refer to Jamieson, Hedges [21].

### Measures

#### Sociodemographic characteristics

Sociodemographic characteristics were assessed at the SAABC study baseline. Mothers self-reported their age, education level (response options: no schooling, primary school, high school, trade or TAFE, university), employment (job, Centrelink, other) and postcode. Socioeconomic position was measured with the Index of Relative Socio-Economic Advantage and Disadvantage (IRSAD), which was calculated based on the participants’ postcode at baseline. The IRSAD summarises information about the economic and social conditions of households within an area and is derived from indicators such as household income, employment, education level, disability and car ownership, among others [22].

#### Primary measures

The Parental-Caregiver Perception Questionnaire (P-CPQ) short-form is a caregiver-informant 8-item measure designed to measure parental/caregivers perception of children’s oral health-related quality of life (COHQoL) [19]. The short-form P-CPQ questions are “How often in the last 3 months because of the condition of their teeth, lips, mouth and jaws has your child”: (1) had pain in the teeth, lips, jaw or mouth; (2) had food caught in or between the teeth; (3) had difficulty biting or chewing firm foods such as fresh apple, corn on the cob or firm meat; (4) taken longer than others to eat a meal; (5) been irritable or frustrated; (6) been upset; (7) not wanted to talk to other children; and (8) missed preschool. From now on, we refer to the P-CPQ items according to their item labels (e.g. “had pain in the teeth, lips, jaw or mouth” is referred to as pain), reported in S1 Table. These 8 items represent 4 conceptual domains: Oral Symptoms (pain and food), Functional Limitation (biting and meals), Emotional Wellbeing (irritable and upset) and Social Well-being (talk and missed). Each item was ranked on 5-point scale ranging from 1 to 5 (1 = Never, 2 = Once or twice, 3 = Sometimes, 4 = Often, and 5 = Every day or almost every day).

The Family Impact Scale (FIS) short-form is a caregiver-informant 8-item measure that evaluates the impact of a child’s oral condition on the entire family life [19]. The short-form FIS questions are “During the last 3 months, because of your child’s teeth, lips, mouth or jaws, how often have you or another family member: (1) been upset; (2) felt guilty; (3) had sleep disrupted; (4) taken time off work (e.g. due to pain, appointments, surgery); (5) had less time for yourself or the family; (6) blamed you or another person in the family; (7) argued with you or others in the family and; (8) required more attention from you or others in the family”. We also refer from now on to the FIS items by their item labels (S1 Table). Each item is ranked on 5-point scale ranging from 1 to 5 (1 = Never, 2 = Once or twice, 3 = Sometimes, 4 = Often, and 5 = Every day or almost every day). These 8 items represent 3 conceptual domains: Parent/Family Activities (*disrupted*, *attention*, *work* and *family*), Parental Emotions (*upfam* and *guilty*) and Family Conflict (*blamed* and *argued*). In both P-CPQ and FIS questionnaires, answers to the “Don’t know” category were recoded as missing.

#### Secondary measures

Overall child well-being was measured through the question “How much is your child’s overall wellbeing affected by the condition of his/her teeth, lips, jaw or mouth?” rated on a 5-point scale ranging from 1 to 5 (1 = Not at all, 2 = Very little, 3 = Some, 4 = A lot, and 5 = Very much). Child oral health was measured through the question “How would you rate your child’s dental health?” rated on a 5-point scale ranging from 1 to 5 (1 Excellent, 2 = Very good, 3 = Good, 4 = Fair, and 5 = Poor).

### Statistical analysis

The statistical analyses were conducted with R packages *EGAnet* [23] and *psychonetrics* [24]. The sample contained 285 mothers with responses to the P-CPQ and FIS regarding 290 children (due to five sets of twins). Since missing values for individual items ranged from 0.3% to 2.8%, missingness was unsubstantial and multiple imputation was not required [25]. Therefore, we employed listwise deletion to records with any missing P-CPQ/FIS item responses (20 mothers with responses regarding 20 children were excluded) and all analyses were conducted with complete cases (n = 265 mothers; 270 children). Percentages were computed to describe the sociodemographic characteristics of the participants.

#### Response categories

Prior to estimating the network models, the first step of the analysis was the investigation of the adequacy of item response categories. To do so, we investigated the distribution of item scores and whether there were response categories that weren’t frequently used. We also investigated potential floor or ceiling effects. Floor/ceiling effects are present when the endorsement of a particular response category exceeds 15% of the expected score under a random (uniform) distribution [26]. For instance, since the P-CPQ and FIS are measured on a 5-point scale, it would be expected that each response category was endorsed by 20% of respondents due to chance alone, so the endorsement of any category from more than 35% of respondents (20% + 15%) indicates potential floor/ceiling effects.

#### Network estimation

To estimate the network models, the Gaussian Graphical Model (GGM) was employed. In the GGM, the nodes represent items and edges represent the structure of conditional dependence between items, calculated as partial correlations coefficients. Considering that the CPQ items are ordinal (with categories ranging from “Never” to “Often”), the input was the polychoric correlation matrix and the network models were estimated with the Graphical Least Absolute Shrinkage and Selection Operator (GLASSO) [23]. The network was plotted with the Fruchterman-Reingold algorithm, which spatially arranges more closely nodes that are strongly associated.

#### Item redundancy

The second step of the analysis was the evaluation of item redundancy. A network should be composed of autonomous causal components. However, when two items are too similar in content, they can be measuring the same causal component and, therefore, are not unique. To quantify the redundancy between nodes, we employed the weighted topological overlap (wTO) statistic with an adaptive alpha [27]. The wTO statistic indicates how similar are the connections of one node in the network (edges) with the connections established by another node. That is, the wTO statistic measures the extent to which two nodes share the same weighted connections (edges) in the network [28, 29]. Redundant items, which exhibited a strong and significant wTO, were combined only when there was theoretical justification for the observed redundancy [30].

#### Exploratory Graph Analysis (EGA)

Once it was established that the network was composed of unique components (i.e. no redundancy), the next step was to employ EGA to evaluate the number of dimensions. We employed EGA using the *walktrap* community-detection algorithm [31, 32] and the number of random walks was optimised using the Total Entropy Fit Index (TEFI) [33]. Since the number of dimensions identified in the sample is subject to sampling variation, to evaluate the robustness of the EGA identified solution, we employed EGA to 1,000 parametric bootstrap samples and examined the *structural consistency* of the EGA-identified dimensions [34]. The *structural consistency* refers to the proportion of times each EGA-identified dimension was *exactly* replicated across the bootstrap samples. Replication across 75% or more bootstrap samples is considered to indicate adequate *structural consistency* [35]. We also evaluated: (1) *item stability*, which is the proportion of times that each item clustered in their EGA-identified dimension [28]; and (2) *network loadings*¸ calculated as the standardized sum of connections of each node within a particular dimension. *Item stability* was also considered adequate when the item clustered more than 75% of the time in the EGA-identified dimension. Network loadings indicate the contribution of each item to the emergence of a coherent dimension in the network. Network loadings can be small (0.00–0.15), moderate (0.16–0.25), or large (0.26–0.35). Network loadings stronger than 0.35 correspond to factor loadings stronger than 0.70 [36].

#### Model fit

The next step was the evaluation of the fit of the network model. We evaluated model fit according to recommendations from Kan, de Jonge [37]. We evaluated the absolute fit of the network model, indicating the degree of correspondence between the model and the data, using traditional fit indices such as the Root Mean Squared Error of Approximation (RMSEA) and Comparative Fit Index (CFI). We followed the conventional guidelines of good model fit indicated by CFI *≥* 0.96 and RMSEA *≤* 0.5 and unacceptable fit indicated by RMSEA ≥ 1.0.

#### Internal consistency reliability

Internal consistency reliability was calculated with McDonald’s coefficient **Ω** [38]. The McDonald’s coefficient **Ω** has two advantages over other coefficients such as Cronbach’s α: (1) it does not rely on assumptions of tau-equivalence; and (2) congeneric model without correlated uniqueness (Dunn, Baguley, & Brunsden, 2014). Internal consistency reliability above 0.70 is considered adequate for research purposes [39].

#### Criterion validity

We examined concurrent validity between the subscales scores with measures of child overall well-being and oral health. It is expected that higher scores on the P-CPQ and FIS (indicating worse COHQoL) would be positively associated with poor child overall well-being and poor child oral health. To investigate these associations, we employed a “risk factor” approach, aiming to identify the children with the highest risks (i.e. poorest COHQoL scores and poorest overall well-being/oral health) [40]. To do so, we dichotomised the criterion variables (overall well-being and oral health) and the subscale scores according to the median. Despite the median split, due to the right skewness of distributions, the low risk (n = 216) and high risk (n = 47) groups of children regarding overall well-being (n = 263 valid responses), and the low risk (n = 183) and high risk (n = 85) groups of children regarding oral health (n = 268 valid responses) had an imbalanced number of individuals. That is, approximately 18% of all children had poor overall well-being and approximately 32% of all children had poor oral health. We employed generalised linear models with log-poisson link to estimated Risk Ratios (RRs) between “low”/“high” subscale scores and “low”/“high” overall well-being (or oral health).

Additionally, we examined whether the subscales scores (without dichotomisation) could predict the occurrence of poor child overall well-being and poor child oral health. Since the majority of children did not have poor overall well-being and/or oral health (i.e. class-imbalanced data), discrimination measures that consider “true negatives”, such as the Area Under the Receiver Operating Characteristic Curve (AUROC), provide an overly optimistic view of the prediction model performance. In the case of class-imbalanced data, the Area Under the Precision Recall-Curve (AUPRC) should be preferred [41]. The AUPRC indicates, across the range of classification thresholds, the average proportion of true positives (e.g. poor oral health) identified among those predicted to be positives (e.g. predicted poor oral health based on the subscale scores). For an in-depth discussion about the AUPRC, please see Ozenne, Subtil [42].

## Results

The participants’ sociodemographic characteristics at the study baseline are displayed in Table 1. The findings indicate that over half of mothers were aged 14 to 24 years, and around 67% had high school or less educational attainment. Around 82% of the mothers were unemployed and more than half lived in the most disadvantaged IRSAD quintile, indicating a mostly disadvantaged population.

**Table 1:** Sociodemographic characteristics of the participants.

Characteristics at study baseline	Mothers with complete responses to the P-CPQ/FIS at the 2-year-follow-up (n = 265)
**Maternal age**	
14–24 years	126 (51.0)
25+ years	121 (49.0)
Missing	18
**Education**	
High school or less	176 (67.2)
Trade or University	86 (32.8)
Missing	3
**Employment**	
Yes	47 (17.9)
No	215 (82.1)
Missing	3
**IRSAD**	
1^st^ (most disadvantaged)	146 (55.8)
2nd	40 (15.3)
3rd	64 (24.4)
4th	9 (3.4)
5^th^ (most advantaged)	3 (1.1)
Missing	3

Note. Numbers and percentages are reported.

### Response categories

**Fig 1:**
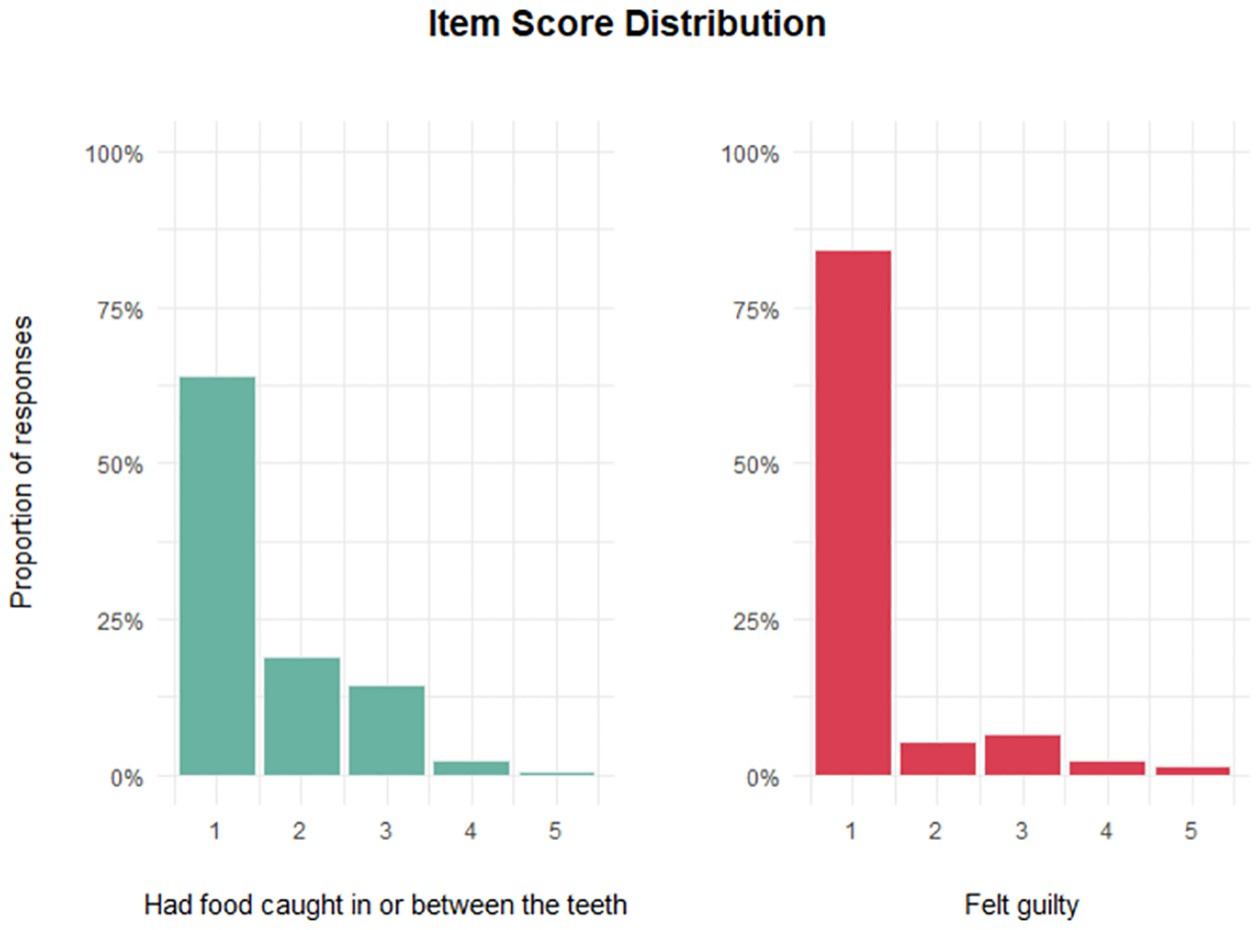
Item score distribution. Note. The x-axis indicates the item response categories ranging from 1 to 5 (1 = Never, 2 = Once or twice, 3 = Sometimes, 4 = Often, and 5 = Every day or almost every day). The y-axis indicates the proportion of respondents that endorsed each response category.

The analysis of the adequacy of response categories from the P-CPQ and FIS indicated strong floor effects across all items. Endorsement of the category “Never” ranged from 64% for the item *food* to 98% on the item *missed*. In addition, the endorsement of the categories “Often” and “Every day or almost every day” was very low. As an example, Fig 1 shows the score distribution of two items, the items *food* from the P-CPQ and *guilty* from the FIS.

It is possible to see in Fig 1 the floor effects and the infrequent endorsement of the “Often”/“Every day or almost every day” categories across the two example items. Due to the low endorsement of the “Often”/“Every day or almost every day” categories across all P-CPQ and FIS items, these categories were combined with the “Sometimes” category, resulting in a 3-point scale for both instruments.

### Item redundancy

The redundancy analysis indicated no redundancy between the items since the wTOs between item pairs were weak and the strongest redundancies were not theoretically meaningful. For instance, the stronger wTO was between the items *biting* and *meals* (wTO = 0.149, p = 0.016). While the items *biting* (“had difficulty biting or chewing firm foods such as fresh apple, corn on the cob or firm meat;”) and *meals* (“taken longer than others to eat a meal”) have conceptual similarities, they represent different causal components since children can take more time to eat a meal not necessarily due to difficulties related to biting or chewing. For instance, the child may be experiencing pain on the lips or behavioural difficulties, leading the child to take longer to eat a meal but there are no issues with biting or chewing. Since no meaningful redundancies were found, we proceeded to estimate the network.

### Network estimation and dimension stability

**Fig 2:**
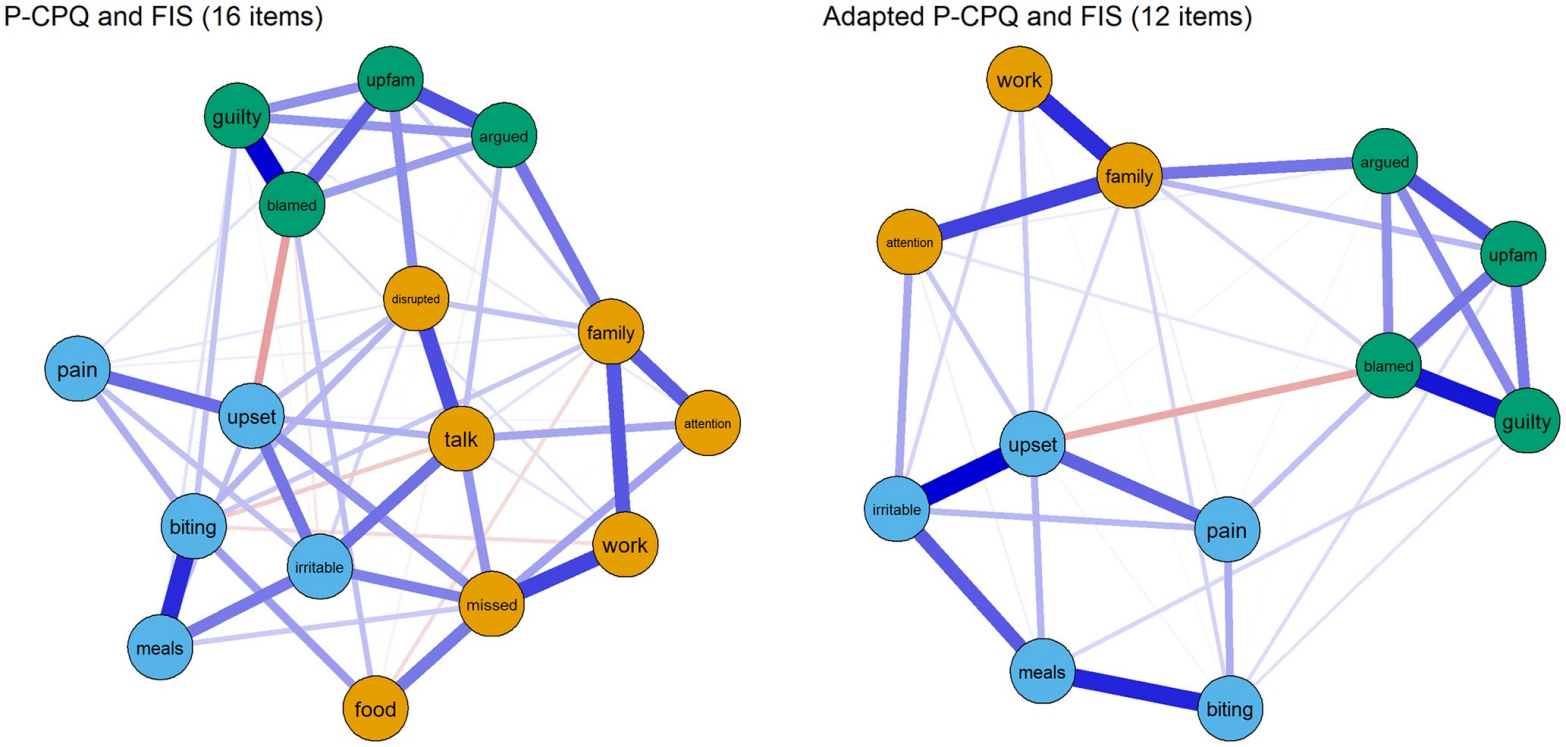
Network of the P-CPQ and FIS items. Note. Positive edges are displayed as blue lines and negative edges are displayed as red lines. Edge weights are represented by the thickness and saturation of the edges. The nodes are coloured according to their EGA-identified dimension.

The network of the P-CPQ and FIS was estimated and EGA identified 3 dimensions. The network of the P-CPQ and FIS and EGA-identified dimensions is displayed in Fig 2 (left column).

**Fig 3:**
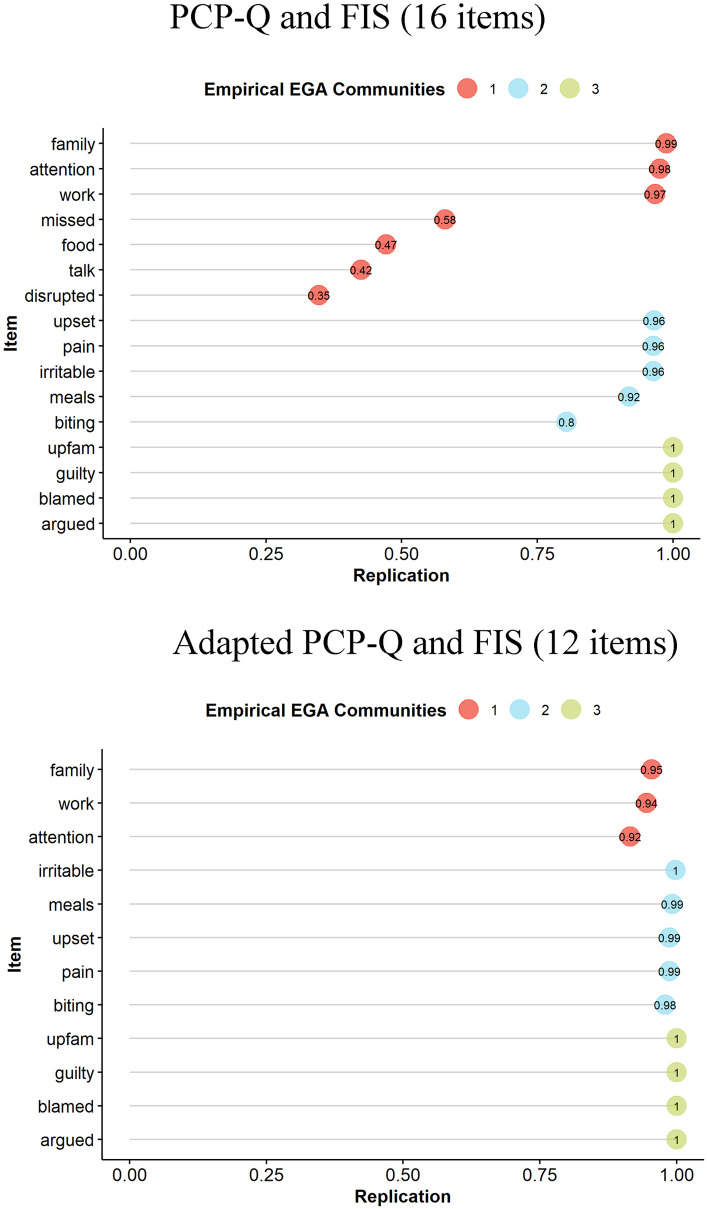
Item stability of the P-CPQ and FIS items. Note. The y-axis indicates the items. The circles are coloured according to their EGA-identified dimension. The x-axis indicates the proportion of times the item clustered with the EGA-identified dimension across the bootstrap samples. The number inside the circle indicates the proportion of times the item clustered with the EGA-identified dimension for each individual item.

The EGA indicated 3 dimensions. While the structural consistency of Dimensions 2 (78%) and 3 (100%) were adequate, the structural consistency of Dimension 1 was poor (23%). The stability of all items is displayed in Fig 3. The investigation of *item stability* showed that low stability was found among the items *disrupted* (33%), *talk* (40%), *food* (48%) and *missed* (60%) (Fig 3, top chart).

To increase the structural consistency of the P-CPQ and FIS, these four items were excluded. After the exclusion of these 4-items, the network model was re-applied and EGA identified again 3 dimensions. This time the structural consistency of all dimensions, Dimension 1 (92%), Dimension 2 (95%) and Dimension 3 (100%), was excellent. Item stability was also excellent (>93%) across all items. Dimension 1 (“orange nodes”) was equivalent to 3 items of the original FIS dimension “Parent/Family Activities”, so it was interpreted as the “Parent/Family Activities” dimension. Dimension 2 (“blue nodes”) was composed only of P-CPQ items (the 5 items P-CPQ retained after the items with low stability were excluded) so it was interpreted as “Children Oral Health-Related Quality of Life (COHQoL)”. Dimension 3 (“green nodes”) was equivalent to the combination of the original FIS dimensions “Parental Emotions” and “Family Conflict”, so it was interpreted as the “Family Conflict” dimension.

### Network loadings

All items displayed large (>0.25) network loadings on their EGA-identified dimensions, except for item *attention* which displayed moderate (>0.15) network loadings. The items *family*, *upset* and *argued* were the only items that displayed moderate cross-loadings (>0.15). The network loadings are displayed in Table 2.

**Table 2:** Network loadings of the child perception questionnaire.

Item	Dimension 3	Dimension 1	Dimension 2
Family	**0.44**	0.08	**0.18**
Work	**0.23**	0.10	0.04
Attention	**0.21**	0.12	0.04
Upset	**0.16**	**0.40**	0.08
Irritable	0.14	**0.40**	0.00
Meals	0.04	**0.37**	0.03
Pain	0.01	**0.25**	0.06
Biting	0.05	**0.25**	0.06
Guilty	0.03	0.06	**0.42**
Blamed	0.05	-0.07	**0.42**
Upfam	0.08	0.08	**0.38**
Argued	**0.17**	0.01	**0.34**

Note. The dimensions were identified by EGA. Network loadings higher than 0.15 (moderate loadings) were highlighted in bold.

### Model fit and reliability

The next step was the evaluation of model fit. The fit indices indicated that the fit of the network model was excellent (χ2(27) = 44.990, p = 0.016, CFI = 0.985, RMSEA = 0.050; 90% CI [0.021, 0.075]). The dimensions of “Parent/Family Activities” (Ω = 0.72; 95% CI [0.58, 0.85]) and “COHQoL” (Ω = 0.78; 95% CI [0.69, 0.86]) displayed adequate reliability, while the dimension of “Family Conflict” (Ω = 0.85; 95% CI [0.80, 0.90]) displayed good reliability.

### Criterion validity

**Fig 4:**
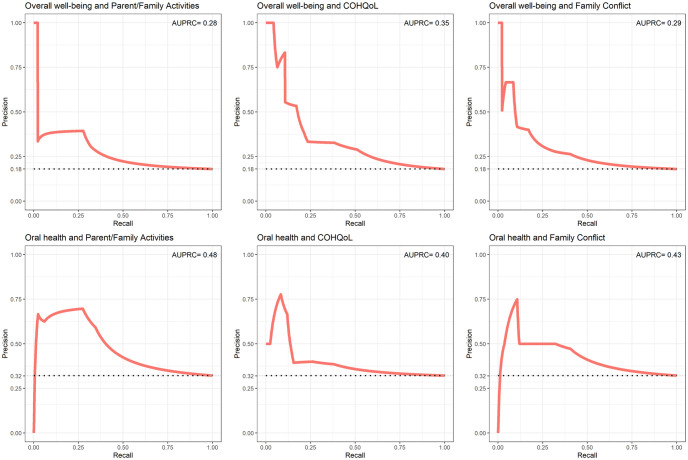
Precision-recall curves between P-CPQ/FIS dimensions and overall well-being/oral health. Note. The y-axis indicates the Precision (i.e. positive predictive value). The x-axis indicates the Recall (i.e. sensitivity). The black dotted line is the prevalence of the outcome in the population (e.g. prevalence of poor overall well-being). The continuous red line is the Precision-Recall Curve (PRC), indicating the precision value according to each recall value. The AUPRC is reported on the top right.

The dimensions of “Parent/Family Activities” (RR = 1.37; 95% CI [1.07, 1.68]), “COHQoL” (RR = 1.20; 95% CI [1.08, 1.32]) and “Family Conflict” (RR = 1.24; 95% CI [1.04, 1.43]) were positively associated with poor overall well-being. For example, children who had high scores on the subscale “COHQoL” (indicating worse COHQoL) had an 20% increased risk of poor overall well-being. Moreover, the dimensions of “Parent/Family Activities” (RR = 1.35; 95% CI [1.13, 1.58]), “COHQoL” (RR = 1.09; 95% CI [0.98, 1.19]) and “Family Conflict” (RR = 1.17; 95% CI [1.02, 1.32]) were also positively associated with poor oral health. To evaluate the performance of the subscale scores regarding predicting poor overall well-being and poor oral health, the AUPRCs are displayed in Fig 4.

Fig 4 indicates that the “Parent/Family Activities” (AUPRC = 0.48; 95% CI [0.42, 0.68]), “COHQoL” (AUPRC = 0.40; 95% CI [0.34, 0.56]) and “Family Conflict” (AUPRC = 0.43; 95% CI [0.37, 0.60]) scores would improve the identification of respondents with poor oral health compared to whether no subscale scores were used and all participants were considered. For example, if an intervention was applied to improve oral health among all children *predicted* to have poor oral health according to their “COHQoL” subscale scores, on average 40% of the children targeted by the intervention would actually have poor oral health and could potentially benefit from the intervention. On the other hand, in case the intervention was applied to every child in the study, only 32% would have poor oral health (instead of 40%). The “Parent/Family Activities” (AUPRC = 0.28; 95% CI [0.23, 0.39]), “COHQoL” (AUPRC = 0.35; 95% CI [0.29, 0.49]) and “Family Conflict” (AUPRC = 0.29; 95% CI [0.24, 0.41]) scores would also improve identification of children with poor overall well-being compared to whether no subscales scores were used to identify the children with poor oral health-related quality of life.

## Discussion

The present study aimed to evaluate the psychometric properties of the short forms of the P-CPQ and FIS for Indigenous children aged 2 to 3 years and their caregivers in South Australia. After the removal of four problematic items, we propose an instrument named Aboriginal Children’s Oral Health-Related Quality of Life Questionnaire (A-COHQoL). This instrument is composed of three dimensions, “Parent/Family Activities”, “COHQoL” and “Family Conflict”. The psychometric properties of the instrument were excellent and the instrument is ready to be applied in future oral health studies with Indigenous children in Australia.

The first step of the analysis was the evaluation of the adequacy of response categories. Our findings indicated that all original P-CPQ and FIS items had strong floor effects; that is, the majority of participants endorsed the category “Never”. For example, 80% of mothers endorsed the “Never” category for the *pain* item, reporting that (up to that point) their children never had pain in the teeth, lips, jaw or mouth. Moreover, the mothers rarely endorsed the categories of “Often” and “Every day or almost every day”. This is possibly due to the low prevalence of oral health problems in children aged 2 to 3 years compared to older children. For example, the original versions of the P-CPQ were developed for older children, aged 6 to 10 years old and 11 to 14 years old [11]. For this reason, considering the age range of our target population (children aged 2 to 3 years), we combined the categories of “Often” and “Every day or almost every day” with the category “Sometimes”. We recommend that the A-COHQoL should be rated on a 3-point scale (1 = Never, 2 = Once or twice, and 3 = Sometimes). Future studies should further investigate whether a 3-point or a 5-point scale is the most appropriate for older Indigenous children.

After the response categories were combined, EGA identified three distinct dimensions. However, the items *disrupted*, *talk*, *food* and *missed* displayed low item stability since these items clustered in some samples with the “COHQoL” items and other samples with the “Parent/Family Activities” items. For example, the item *missed* (“How often has your child had missed preschool?”) was originally from the P-CPQ and was designed to exclusively measure children’s oral health-related quality of life. However, when a child misses school due to oral health problems, most likely it also impacts family activities. Hence, this item was strongly associated with both dimensions and it was not clear whether it should be summed together with the other “COHQoL” items or the “Parent/Family Activities” items. Since the four items with low stability did not belong to a particular dimension (making it conceptually challenging to sum the item score with other items to create a subscale score), the four items with low stability were excluded.

Once the items with low stability were removed, EGA identified again three distinct dimensions and this time these dimensions had excellent structural consistency. These dimensions were named “Parent/Family Activities”, “COHQoL” and “Family Conflict”. The evaluation of model fit showed excellent model fit and internal consistency reliability was adequate for all three dimensions. All 12 items had substantive network loadings on their EGA-identified dimension. Although three items displayed moderate cross-loadings, we concur with Golino, Lillard [35] that these items: “were relatively stable despite having high cross-loadings. This suggests that these items are structurally consistent with their dimension but may be strongly related to items outside of their dimension”. That is, despite the connections with other dimensions, these items were strongly associated and stable (i.e. structurally consistent) with their EGA-identified dimensions. For this reason, these items were retained in the final version of the A-COHQoL.

To the best of our knowledge, this is the first study that validated any of the COHQoL measures for an Indigenous population. It is also the first study to investigate the validity of the P-CPQ and FIS through network psychometrics. The use of modern psychometric methods based on network science (i.e. network psychometrics) brings two theoretical contributions to the COHQoL literature. Firstly, we were able to evaluate the items of the P-CPQ and FIS together. Our findings showed that they configured *three distinct clusters* (i.e. dimensions) of mutually reinforcing behaviours that *belong to a broader connected network* of behaviours related to COHQoL. This was the original intent of the P-CPQ and FIS developers, who envisioned both instruments to work as complementary measures of COHQoL [11, 12]. However, all previous studies that employed traditional psychometric methods, such as factor analytical [43] or item response theory models [44], evaluated the properties of the COHQoL instruments separately (or evaluated the properties of a single CHQoL instrument). Secondly, the network psychometrics framework makes it possible to investigate associations between the instruments (P-CPQ and FIS) at an *item/behavioural level* instead of a *dimension/construct level* [45]. For example, the network model showed that there is a *conditional dependence* between the items *irritable* (“been irritable or frustrated”) and *attention* (“required more attention from you or others in the family”) indicating that children who were more irritable due to oral health problems were also more likely to require attention from the family. Traditional models, such as factor analytical and structural equation models, are commonly used in the COHQoL literature to investigate associations at a *dimension/construct* level. For example, a structural equation model indicated that children of parents with positive rearing practices had better *overall* COHQoL [46]. However, the study did not indicate which specific COHQoL behaviours (as measured by the P-CPQ and FIS items) were impacted by positive rearing practices to elucidate the mechanism behind how parental practices affect overall COHQoL. The network framework enables the investigation of COHQoL behaviours at a system (behavioural level) [47] and can provide new insights to the COHQoL literature.

Our study also had limitations. One limitation was that since our sample size was moderate (although it had a large number of participants considering the recruitment challenges in Indigenous health studies [48]), we decided to not split the sample into *development* (to estimate the network model) and *validation* samples (to evaluate model fit) to ensure the maximum statistical power for the network model estimation. Hence, since model estimation and model fit were conducted on the same dataset, it is possible that the fit of the network model was overestimated [49, 50] and future studies should further confirm the good model fit of the A-COHQoL in other Aboriginal communities.

In conclusion, the A-COHQoL is a psychometrically robust and sensitive instrument that can be appropriate for use among Indigenous Australian child groups. We recommend its implementation among older Indigenous Australian child groups, and among Indigenous child groups in other countries taking into consideration local contexts and needs.

## Supporting information

S1 Table Item content and labels. (DOCX)
