# Supplementary material for: A network psychometric validation of the Children Oral Health-Related Quality of Life (COHQoL) questionnaire among Aboriginal and/or Torres Strait Islander children
Source: PLoS One. 2022 Aug 18;17(8):e0273373. doi: 10.1371/journal.pone.0273373 (PMC9387801; doi:10.1371/journal.pone.0273373)
Supplement: S1 Table — (DOCX) [file pone.0273373.s001.docx]

**S1 Table. Item content and labels.**

| Item wording | Item label |
| --- | --- |
| **P-CPQ** |  |
| **How often in the last 3 months because of the condition of their teeth, lips, mouth and jaws has your child** |  |
| had pain in the teeth, lips, jaw or mouth? | pain |
| had food caught in or between the teeth? | food |
| had difficulty biting or chewing firm foods such as fresh apple, corn on the cob or firm meat? | biting |
| taken longer than others to eat a meal? | meals |
| been irritable or frustrated? | irritable |
| been upset? | upset |
| not wanted to talk to other children? | talk |
| missed preschool? | missed |
| **FIS** |  |
| **During the last 3 months, because of your child’s teeth, lips, mouth or jaws, how often have you or another family member** |  |
| been upset? | upfam |
| felt guilty? | guilty |
| had sleep disrupted? | disrupted |
| taken time off work (e.g. due to pain, appointments, surgery)? | work |
| had less time for yourself or the family? | family |
| blamed you or another person in the family? | blamed |
| argued with you or others in the family? | argued |
| required more attention from you or others in the family? | attention |

Note. The label “upset” refers to the item “How often in the last 3 months because of the condition of their teeth, lips, mouth and jaws has your child been upset?”. The label “upfam” refers to the item “During the last 3 months, because of your child’s teeth, lips, mouth or jaws, how often have you or another family member been upset?”.
